# COVID-19 Stress and Addictive Social Media Use (SMU): Mediating Role of Active Use and Social Media Flow

**DOI:** 10.3389/fpsyt.2021.635546

**Published:** 2021-02-09

**Authors:** Nan Zhao, Guangyu Zhou

**Affiliations:** School of Psychological and Cognitive Sciences, Beijing Key Laboratory of Behavior and Mental Health, Peking University, Beijing, China

**Keywords:** active use, addictive social media use, addiction, COVID-19, disaster stress, social media flow

## Abstract

The ongoing COVID-19 pandemic is likely to enhance the risk of addictive social media use (SMU) as people spend more time online maintaining connectivity when face-to-face communication is limited. Stress is assumed to be a critical predictor of addictive SMU. However, the mechanisms underlying the association between stress and addictive SMU in crises like the current COVID-19 situation remain unclear. The present study aimed to understand the relationship between COVID-19 stress and addictive SMU by examining the mediating role of active use and social media flow (i.e., an intensive, enjoyable experience generated by SMU that perpetuates media use behaviors). A sample of 512 Chinese college students (*M*_*age*_ = 22.12 years, *SD* = 2.47; 62.5% women) provided self-report data on COVID-19 stress and SMU variables (i.e., time, active use, flow, addictive behavior) via an online survey from March 24 to April 1, 2020. The results showed that COVID-19 stress was positively associated with tendencies toward addictive SMU. Path analyses revealed that this relationship was significantly serially mediated by active use and social media flow, with SMU time being controlled. Our findings suggest that individuals who experience more COVID-19 stress are at increased risk of addictive SMU that may be fostered by active use and flow experience. Specific attention should be paid to these high-risk populations and future interventions to reduce addictive SMU could consider targeting factors of both active use and social media flow.

## Introduction

The ongoing global pandemic of COVID-19 caused by a novel coronavirus (SARS-CoV-19) has a significant impact on individual lifestyle. Due to policies to limit the spread of the virus, such as the “shelter-in-place” order (1), people, willing or not, are undergoing a transition from offline to online activities (2). In addition to remote work or remote learning, many people spent increased time on social media (SM), such as Facebook and Twitter, which could satisfy their need for disaster-related information, entertainment as well as interpersonal communication (3, 4). Despite the undeniable advantageous role that SM plays in an emergency like COVID-19 (5), escalations in the use of SM are likely to bring about addictive social media use (SMU). According to Andreassen (6), addictive SMU is defined as excessive and compulsive use of social platforms. As a specific form of Internet addiction, addictive SMU entails six core components of a behavioral addiction model (7) including (1) being unduly concerned with or spending too much time on SM (salience), (2) using SM to regulate negative emotions or forget personal problems (mood modification), (3) feeling an urge to invest more time on SM to attain the same level of pleasure (tolerance), (4) feeling uncomfortable, restless, and irritable when prohibited from SM for a time (withdrawal), (5) causing harm to work, life and interpersonal relationship due to SMU (conflict), (6) trying to give up SMU but cannot manage it (relapse). It should be noted that additive SMU has not been formally recognized as a psychiatric disorder, though its definition is in line with diagnostic addiction criteria (8). Recent studies have suggested the increased tendency of Internet addiction following the pandemic onset (9, 10). However, little is known about the influence of COVID-19 on the development of addictive SMU as well as the underlying mechanisms.

People often resort to media use in response to daily hassles and stressful life events (11–13). In their stress and coping theory, Lazarus and Folkman (14) differentiated two types of coping strategies that people normally adopted to manage stress. One is problem-focused coping (i.e., engage in behaviors that could help solve problems) and the other is emotion-focused coping (i.e., regulate emotional responses to the problem without affecting the actual presence of stress). When confronted with challenges created by COVID-19, people are likely to turn to SM for both problem-focused coping (e.g., browsing health-related information) and emotion-focused coping (e.g., venting emotions for mood management, joining online communities for social support) (15). SM also promoted collective coping by becoming a venue for survivors to express feelings, document traumatic events, and reconstruct meaning in the aftermath of natural disasters (16). However, the reliance on SM for coping is not only associated with benefits. For example, recent research described that increased Internet use when coping with stress posed by the COVID-19 pandemic did not effectively enhance well-being among older adults (17). Although trauma-induced stress could be temporarily alleviated by certain online activities, it has the potential to lead to excessive SMU. Both cross-sectional and longitudinal studies have established a positive link between daily stress and addictive Facebook use (12, 18, 19). So far, however, there has been little discussion about the relationship between disaster-specific stress and addictive SMU. Along with the above theories and findings, it is therefore hypothesized that people who experience greater stress related to COVID-19 are at greater risk of addictive SMU.

Active use is a potential mediator explaining the effect of COVID-19 related stress on addictive SMU. Active use refers to activities that facilitate direct exchanges with others (e.g., commenting on posts of friends, tagging, “liking,” posting a status update, sharing pictures or videos), while passive use involves activities, such as browsing news feeds or viewing posts of others without any direct exchanges (20). By differentiating the two types of SM activities, prior research suggested that active use could be beneficial in terms of enhancing social connectedness, subjective well-being and reducing loneliness (21–23). However, active use could be excessive when it is motivated to compensate for psychosocial problems (24). Following the theory of basic psychological needs (25), it might be possible that individuals who experience considerable stress related to COVID-19 (e.g., infection, quarantine) may feel that their basic psychological needs (i.e., autonomy, capacity, and relationships) are not satisfied and thus turn to active use of SM to compensate for their unmet needs. On the other hand, active SMU, such as broadcasting has been proved to be positively associated with addictive Facebook use (26). However, there are no studies that directly tested the mediating role of active use in the relationship between COVID-19 stress and addictive SMU.

Flow could be another antecedent of addictive SMU. Flow is a concept of positive psychology, which refers to a state of concentration that is so focused that people find themselves deeply absorbed in that activity (27). The state of flow is intrinsically self-reinforcing, in which people can experience feelings of joy, pleasure, and satisfaction and therefore can be motivated to repeat the ongoing activities (28). Researchers integrated the concept of flow into online activities (29). Specifically, Kwak et al. (30) proposed six elements to characterize the flow experience on SM: focused attention (i.e., high concentration on SM), enjoyment (i.e., pleasant experience due to SMU), curiosity (i.e., desire to know things happened on social media), telepresence (i.e., feeling the world created by SM is real), time-distortion (i.e., loss of a sense of time during SMU) and self-disclosure (i.e., revelation of personal information during SMU). In the media context, it has been suggested that flow experience resulted from repetitive behaviors through a desire to maintain positive feelings could raise the frequency and intensity of media consumption, and therefore, results in addictive behaviors (31). In line with this notion, previous studies proved that flow was a positive predictor of Internet addictive symptoms (32), Internet gaming disorder (33), and addictive Facebook use (34). Therefore, it seems plausible to hypothesize that flow is positively associated with addictive SMU.

As reviewed above, both active use and SM flow are associated with addictive SMU. Moreover, it is suggested that flow appears when people are engaged in SMU activities with characteristics of social interaction, such as communicating with others and receiving instant feedback (35). Therefore, it is reasonable to posit that SM flow mediates the relationship between active use and addictive SMU (i.e., active use → SM flow → addictive SMU). Previous studies on narcissistic individuals indicated that this pathway might possibly exist. Brailovskaia and Margraf (21) found that narcissistic individuals, driven by a need for self-representation, actively engaged in SM (e.g., uploading attractive photos, writing updates, and joining online discussion groups) to maintain a positive impression. However, this process involving active use further contributed to the risk of Facebook addiction through increasing flow experience (36). In the context of COVID-19, one of the antecedents of active use might be COVID-19 stress given that people who experienced more disaster-related stressful events may resort to active SMU for coping (15). Therefore, it is reasonable to assume that the serial mediation effect of active use and flow may exist between the relationship of COVID-19 stress and addictive SMU (i.e., COVID-19 stress → active use → SM flow → addictive SMU). However, this underlying mechanism has not been empirically tested to date.

**Figure 1:**
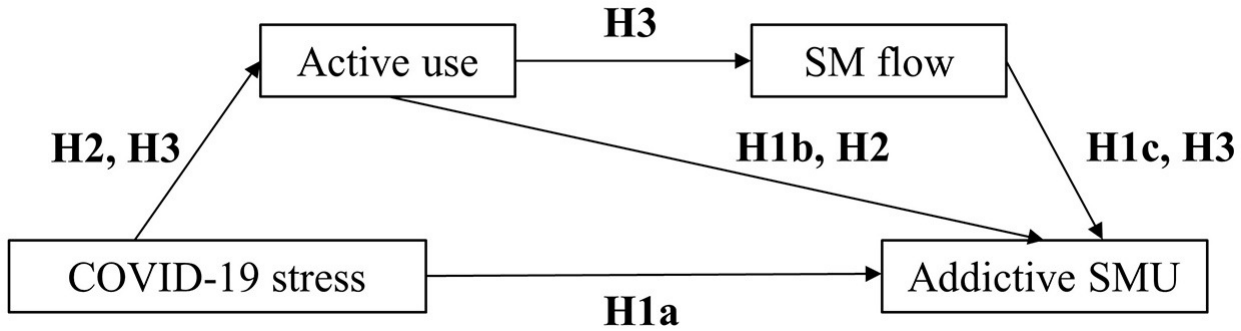
The hypothesized model concerning the relationship between COVID-19 stress and addictive SMU: active use and SM flow as serial mediators.

The present study aims to clarify the relationships between COVID-19 stress, active use, SM flow, and addictive SMU. Figure 1 illustrated the hypothesized model. To be specific, it is hypothesized that COVID-19 stress (Hypothesis 1a), active use (Hypothesis 1b), and flow (Hypothesis 1c) are all positively associated with addictive SMU; active use mediates the relationship between COVID-19 stress and addictive SMU (Hypothesis 2); active use and flow sequentially mediate the relationship between COVID-19 stress and addictive SMU (Hypothesis 3).

## Method

### Participants and Procedure

The study was approved by the Institutional Review Board of Peking University. From March, 24, to April, 01, 2020, an advertisement of the study was posted on Wechat, one of the most popular SM platforms in China. The post was shared and reposted hundreds of times. People who were willing to join the study could scan the quick response code on the poster, which directed them to online informed consent. Participants then spent ~10 min to complete an online questionnaire via www.sojump.com.

**Figure 2:**
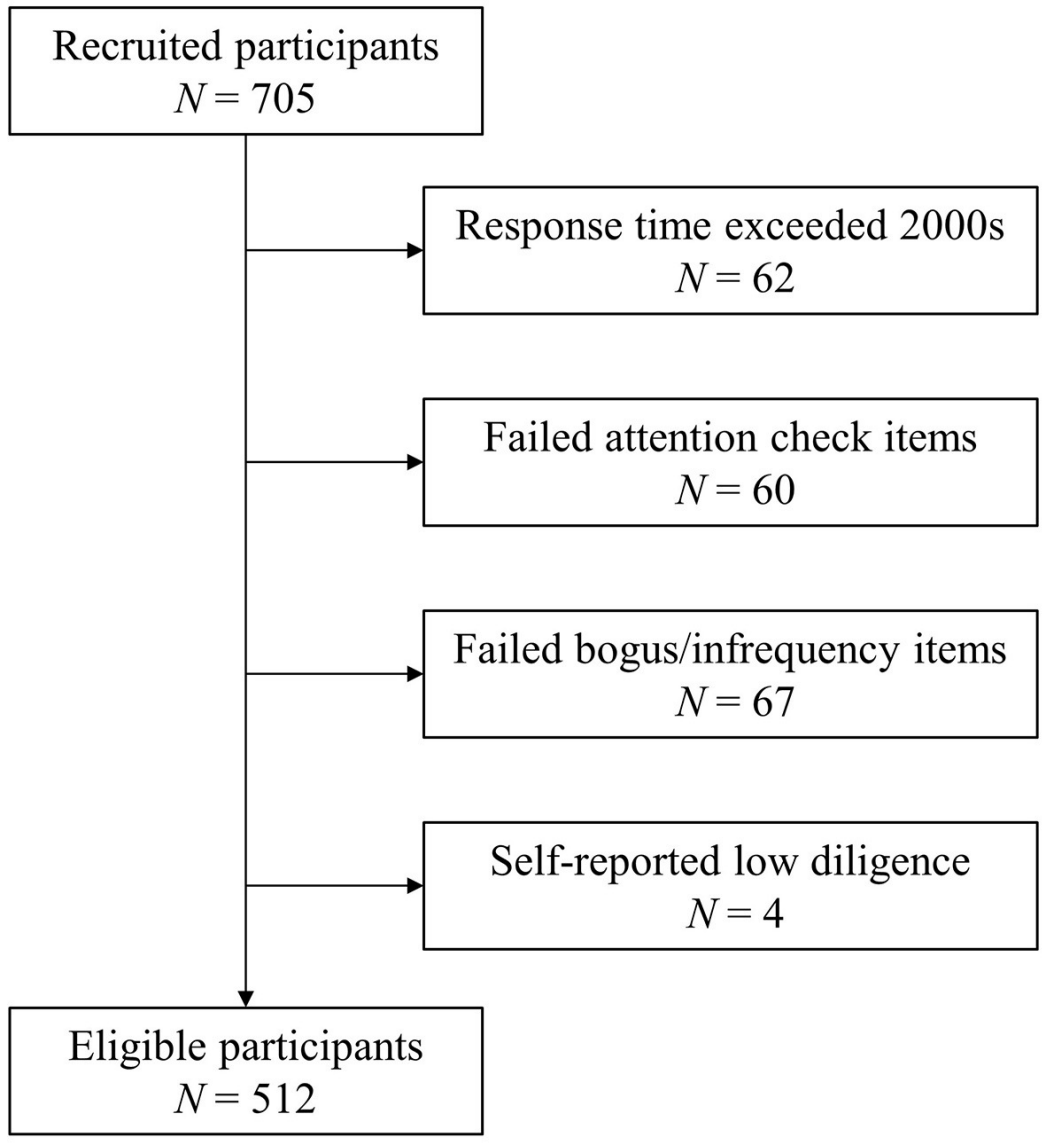
Flow chart showing the steps involved in establishing the study sample.

A total number of 705 college students volunteered to participate in the study and completed the questionnaire. Following the recommendations of Curran (37), data of 192 participants were identified as invalid and removed before normal analysis (see Figure 2). The exclusion criteria included: (1) spent more than 2,000 s on the questionnaire (*N* = 62); (2) failed at least one of two attentional check items (e.g., “please answer with ‘agree’”; *N* = 60); (3) failed at least one of two bogus items (e.g., “I have never used a mobile phone in my life,” *N* = 67); (4) self-reported low diligence at the end of the questionnaire (e.g., “In your honest opinion, should we use your data in our analyses?”; *N* = 4). The final sample comprised of 512 college students. The age of the sample ranged from 18 to 30 years (*M*_age_ = 22.12, *SD* = 2.47). Most of the participants were female (*N* = 320, 62.5%) and of Han ethnic (*N* = 480, 93.8%). As for the educational attainment, 58% (*N* = 297) of the participants obtained a bachelor degree, 32.4% (*N* = 166) obtained a master degree and 9.5% (*N* = 49) obtained a doctor degree.

### Measures

#### COVID-19 Stress

Adapted from the SARS-related stress by Main et al. (38), a checklist of ten items was used to assess participants' experience of COVID-19 related stressful events. Participants were asked whether or not they (1) confirmed or suspected infection; (2) experienced loved ones dying from infection; (3) witnessed others dying from infection; (4) worked with infectious patients; (5) volunteered for the disease prevention and control; (6) lacked food; (7) lacked masks or disinfectants; (8) had no access to medical care; (9) experienced the lockdown of Wuhan city; (10) stayed alone for a long time. Responses were “yes” (coded as 1) or “no” (coded as 0). The total number of events endorsed was computed to reflex the indexes of COVID-19 stress. The composite score ranged from 0 to 10, with a higher score indicating a higher level of COVID-19 stress.

#### Addictive Social Media Use

Addictive SMU was measured with the brief version of Bergen Facebook Addiction Scale [BFAS; (39)]. The 6-item scale assessed addictive SMU behaviors in six aspects (i.e., salience, mood modification, conflict, withdrawal, relapse, tolerance) with a 5-point Likert scale (1 = very rarely, 5 = very often). An example item is “How often do you become restless or troubled if you have been prohibited from using SM?” The sum score ranged from 6 to 30, with a higher score indicating a higher level of addictive SMU (Cronbach's α = 0.84).

#### Active Use

Active SMU was measured with four items adapted from the assessment tool developed by Brailovskaia and Margraf (21). Participants were instructed to answer how often they engaged in each of four activities on SM since the COVID-19 outbreak on a 4-point Likert scale (0 = never, 3 = very often). Activities included: (1) updated status (including texts, photos, or short videos) about one's own life; (2) updated status about the COVID-19 pandemic; (3) liked, commented, or shared others' update; (4) liked, commented, or shared news about the COVID-19 pandemic. The sum score ranged from 0 to 12, with a higher score indicating a higher level of active SMU (Cronbach's α = 0.78).

#### Social Media Flow

Flow experience related to SMU was assessed with a modified version of “Facebook flow” developed by Brailovskaia et al. (34). The scale included eleven items that captured five core aspects of flow experience (i.e., focused attention, enjoyment, curiosity, telepresence, and time-distortion). Items were rated on a 5-point Likert scale (1 = totally disagree, 5 = totally agree). An example item is “While using social media, I'm deeply engrossed.” The sum score ranged from 11 to 55, with a higher score indicating a higher extent of SM flow (Cronbach's α = 0.82).

#### Covariate

*SMU time* was measured following the method of Lin et al. (40). Participants were instructed to recollect how many hours per day they spent on each of six widely-used SM platforms in China (i.e., Weibo, Wechat, Douyin, Kuaishou, Douban, Zhihu) during the period of severe pandemic (i.e., 20 January to 16 February 2020, characterized by a sharp increase in the number of infected from 258 to 70,635). Responses ranged from 0 to 12 h for each platform. Time spent on each platform per day was summed up to reflect the total daily hours of SMU time, with more hours indicating a higher level of SM consumption.

### Statistical Analyses

Data were analyzed with SPSS 22.0 and PROCESS macro (41). First, descriptive statistics and correlations between the main variables were conducted. Second, to examine the relationship between COVID-19 stress and addictive SMU, a serial mediation was performed with COVID-19 stress as the independent variable, active use and SM flow as mediators in sequence, and addictive SMU as the dependent variable. The bootstrapping procedures in the Model 6 of PROCESS macro was used to test the significance of the serial indirect effects, with 5,000 times of random sampling. A 95% confidence interval (CI) of indirect effect that did not contain zero indicated a significant mediation effect at the 0.05 level. A reverse mediation model in which SM flow and active use interchanged positions was also tested.

## Results

### Preliminary Analyses

Table 1 presented the demographics and responses of participants. Descriptive statistics and correlations between the main variables are presented in Table 2. Variables showed a univariate normal distribution with the skewness and kurtosis ranging from −2 to 2. COVID-19 stress was significantly positively correlated with active use, SM flow, and addictive SMU. Active use was significantly positively correlated with SM flow and addictive SMU. SM flow was significantly positively correlated with addictive SMU. SMU time was significantly positively correlated with active use, SM flow, and addictive SMU, while COVID-19 stress was not significantly correlated with SMU time.

**Table 1:** Demographics and responses of participants (*N* = 512).

**Variables**	***N* (*M*)**	**% (*SD*)**
Age	22.12	2.47
Gender (female)	320	62.5
Ethnic (Han)	480	93.8
**Educational attainment**		
Bachelor degree	297	58.0
Master degree	166	32.4
Doctor degree	49	9.5
**COVID-19 stress**		
Self or a close other confirmed or suspected COVID-19 infection	2	0.4
Experienced loved ones dying from COVID-19	1	0.2
Witnessed others dying from COVID-19	7	1.4
Worked with infectious patients	22	4.3
Volunteered for the disease prevention and control	77	15.0
Lacked food	43	8.4
Lacked face masks or disinfectants	326	63.7
No access to medical care	12	2.3
Experienced the lockdown of Wuhan city	15	2.9
Stayed alone for a long time due to COVID-19	167	32.6
**SMU Time (h/day)**		
Weibo	1.17	1.15
Wechat	1.48	1.36
QQ	0.42	0.82
Douban	0.11	0.48
Zhihu	0.47	0.74
Douyin	0.34	0.92
Kuaishou	0.07	0.39

*SMU, social media use*.

**Table 2:** Descriptive statistics and correlations between the main variables (*N* = 512).

**Variables**	**1**	**2**	**3**	**4**	**5**	**6**	**7**
1. Gender	—						
2. Age	0.01	—					
3. COVID-19 stress	0.03	−0.08	—				
4. Active use	−0.06	−0.08	0.15^**^	—			
5. SM flow	0.04	−0.03	0.13^**^	0.28^***^	—		
6. Addictive SMU	0.05	−0.05	0.16^***^	0.25^***^	0.46^***^	—	
7. SMU time	−0.05	−0.04	0.03	0.12^**^	0.15^**^	0.13^**^	—
Range	0–1	18–30	0–5	0–12	17–55	6–30	2–34
*M*	0.38	22.12	1.31	5.33	35.79	16.52	10.44
*SD*	0.49	2.47	0.94	2.88	6.49	5.44	5.61
Skewness	0.52	0.39	0.60	0.18	−0.05	0.11	1.22
Kurtosis	−1.74	−0.24	0.47	−0.35	0.14	−0.58	1.69

*Gender coded as 0 = female, 1 = male; SM, social media; SMU, social media use; M, mean; SD, standard deviation*;

** *p < 0.01*,

*** *p < 0.001*.

### Serial Mediation Analyses

**Figure 3:**
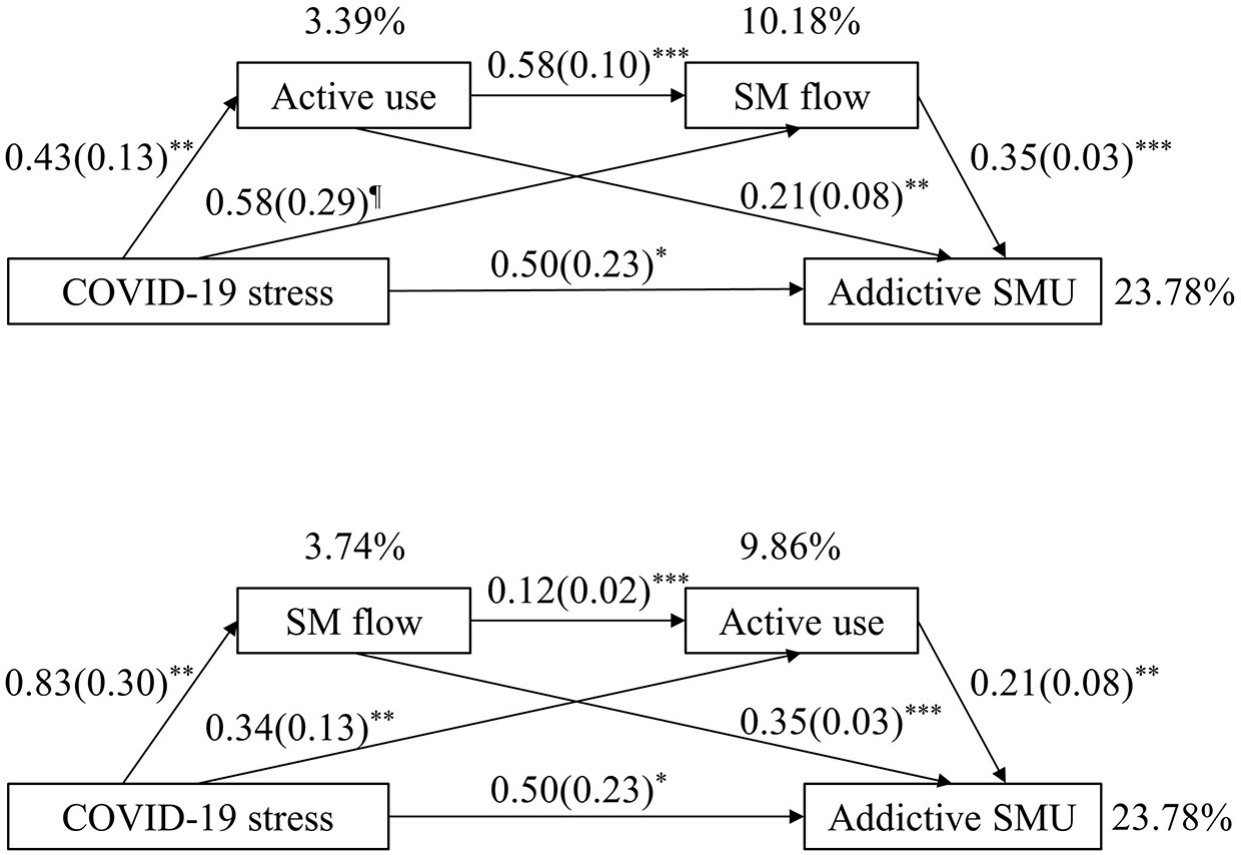
Mediation model of the effect of COVID-19 stress on addictive SMU with active use and SM flow as mediators (*N* = 512). SM, social media; SMU, social media use. SMU time was controlled in the model as a covariate. Unstandardized coefficients and standard errors (SE) were reported. Percentages indicate the explained variance of each mediator and dependent variable in the model. All path coefficients were significant. ^¶^*p* = 0.049, *p < 0.05, **p < 0.01, ***p < 0.001.

To investigate the relationship between COVID-19 stress and addictive SMU, serial mediation analysis was conducted with active use and SM flow as mediators using Model 6 of Hayes' PROCESS tool. Results are summarized in Figure 3 and Table 3. Total effects of COVID-19 stress on addictive SMU was significant (*b* = 0.88, *SE* = 0.25, *p* < 0.001, 95%CI [0.3883, 1.3721]). The direct paths from COVID-19 stress to active use (*b* = 0.43, *SE* = 0.13, *p* = 0.001, 95%CI [0.1720, 0.6949]) and SM flow (*b* = 0.58, *SE* = 0.29, *p* = 0.049, 95%CI [0.0018, 1.1486]) were also significant. Meanwhile, the direct effects from mediators, namely active use (*b* = 0.21, *SE* = 0.08, *p* = 0.007, 95%CI [0.0590, 0.3619]) and SM flow (*b* = 0.35, *SE* = 0.03, *p* < 0.001, 95%CI [0.2776, 0.4125]) to addictive SMU were significant. Moreover, the path from the first mediator (active use) to the second mediator (SM flow) was significant (*b* = 0.58, *SE* = 0.10, *p* < 0.001, 95%CI [0.3920, 0.7700]). The indirect effect tests were significant for the first mediator (active use indirect *b* = 0.09, *SE* = 0.05, 95%CI [0.0129, 0.2047]), the second mediator (SM flow indirect *b* = 0.20, *SE* = 0.10, 95%CI [0.0052, 0.3961]), and both mediators in sequence (*b* = 0.09, *SE* = 0.03, 95%CI [0.0287, 0.1594]). When two mediators were included in the model, the direct effect of COVID-19 stress on addictive SMU was still significant (*b* = 0.50, *SE* = 0.23, *p* = 0.027, 95%CI [0.0581, 0.9491]), with active use, SM flow and addictive SMU accounted for 3.39, 10.18, and 23.78% of the total variance, respectively.

**Table 3:** Decomposition of the effect of COVID-19 stress on addictive SMU (*N* = 512).

	**Mediation model**				**Reverse mediation model**			
	**β**	***b***	***SE***	**95%CI**	**β**	***b***	***SE***	**95%CI**
**Direct effect**								
COVID-19 stress	0.09	0.50	0.23	(0.0581, 0.9491)	0.09	0.50	0.23	(0.0581, 0.9491)
Active use	0.11	0.21	0.08	(0.0590, 0.3619)	0.11	0.21	0.08	(0.0590, 0.3619)
SM flow	0.41	0.35	0.03	(0.2776, 0.4125)	0.41	0.35	0.03	(0.2776, 0.4125)
**Indirect effects through**								
Active use	0.02	0.09	0.05	(0.0129, 0.2047)	0.01	0.07	0.04	(0.0077, 0.1681)
SM flow	0.03	0.20	0.10	(0.0052, 0.3961)	0.05	0.29	0.10	(0.0850, 0.4908)
Active use and SM flow	0.02	0.09	0.03	(0.0287, 0.1594)	–			
SM flow and active use	–				0.004	0.02	0.01	(0.0023, 0.0475)

*SMU, social media use; SM, social media*.

For the alternative reverse mediation model, the direct effects of COVID-19 stress, SM flow and active use on addictive SMU were exactly the same as those of original mediation model. The direct paths from COVID-19 stress to SM flow (*b* = 0.83, *SE* = 0.30, *p* = 0.006, 95%CI [0.2400, 1.4139]) and active use (*b* = 0.34, *SE* = 0.13, *p* = 0.009, 95%CI [0.0834, 0.5928]) were significant. Moreover, the path from the first mediator (SM flow) to the second mediator (active use) was significant (*b* = 0.12, *SE* = 0.02, *p* < 0.001, 95%CI [0.0778, 0.1528]). The indirect effect tests were significant for the first mediator (SM flow indirect *b* = 0.29, *SE* = 0.10, 95%CI [0.0850, 0.4908]), the second mediator (active use indirect *b* = 0.07, *SE* = 0.04, 95%CI [0.0077, 0.1681]), and both mediators in sequence (*b* = 0.02, *SE* = 0.01, 95%CI [0.0023, 0.0475]). SM flow, active use and addictive SMU accounted for 3.74, 9.86, and 23.78% of the total variance of the model, respectively.

## Discussion

The present study examined the relationship between COVID-19 stress, active use, SM flow, and addictive SMU in a sample of Chinese college students. Consistent with Hypothesis 1a, COVID-19 related stress was associated with a greater tendency of addictive SMU, higher level of active use, and SM flow experience. Both active use and SM flow were directly related to addictive SMU, confirming Hypothesis 1b and 1c. Consistent with Hypothesis 2, a mediating effect of active use was found between COVID-19 stress and addictive SMU. In addition, the present findings demonstrate that active use and SM flow in sequence mediate the relationship between COVID-19 stress and addictive SMU, confirming Hypothesis 3. However, the reverse mediation model with SM flow as the first mediator and active use as the second was also significant, which is contrary to our hypotheses. To sum up, the significant results in part confirmed our hypotheses and therefore allow a better understanding of why people who suffer from pandemic-related stress are at enhanced risk to develop addictive SMU.

Prior research suggests that stress and addictive SMU are positively related (12). Stress was considered to be a common risk factor of both chemical addictions [e.g., drug dependence; (42)] and behavioral addictions [e.g., excessive smartphone use; (43)]. Earlier studies found perceived daily stress to be positively related to addictive Facebook use (18) and Internet addiction (44). The current study adds value to the existing literature by measuring objective pandemic-related stress rather than subjectively perceived stress. The present results prove a positive association between stress and addictive SMU, which is in line with previous results (45). Given the fact that the prevalence of Internet addiction has increased during the COVID-19 pandemic (9, 10), it is imperative to identify individuals who are susceptible to addictive SMU. Our current findings suggest that people who experience extremely stressful events during the epidemic, such as quarantine, infection, or food shortages are at enhanced risk for developing addictive SMU.

Understanding the mediators of the association between COVID-19 stress and addictive SMU is important for identifying risky factors and developing prevention strategies for SM addiction. The current study revealed the mediating role of active use between COVID-19 stress and addictive SMU, which is in accordance with media use theories. According to the use and gratifications theory, people satisfy their unique social and psychological needs by exposure to mass media (46). A recent study suggested that people are motivated to use SM for several purposes, such as searching for information, seeking social interaction, beating boredom and pastime, escaping from negative emotions, and searching for positive emotions (47). When confronted with COVID-19-related stress, individuals are likely to actively engage in SM activities, such as disclosing personal feelings to relieve negative emotions. This behavior is likely to be reinforced since people receive empathetic responses and social support from online interactions, which creates a justification for further checking and posting on SM later on (48). Thus, it can be assumed that active SMU predicts addictive behaviors due to the negative reinforcement of mood alteration (49). Indeed, both empirical studies and meta-analysis suggest that individuals with the wish to escape from negative emotions caused by offline conflicts are at enhanced risk to develop addictive SMU (47, 50). Similarly, by proposing the concept of compensatory Internet use, Kardefelt-Winther (24) suggested that people use the online world to escape real life stress or to alleviate negative mood, which ultimately leads to negative outcomes. Therefore, the significant indirect path from COVID-19 stress to addictive SMU via active use implies that excessive active use acts as a maladaptive coping strategy in the time of the COVID-19 crisis. Another important finding is that active use independently or combined with flow explained the relationship between COVID-19 stress and addictive SMU. Furthermore, flow emerged as a stronger factor accounting for addictive SMU than active use, which is consistent with previous findings that people who experience flow (i.e., immersive pleasure) are particularly prone to behavioral addiction (32, 33).

However, findings from two serial mediation models suggest that the effects between active use and SM flow are likely bidirectional. Indeed, existed evidence on the relationships between SM use and flow is mostly correlational. Social network sites (SNS) flow was found to be predictive of increased SNS self-disclosure, a form of active use to build interpersonal connections (30). Another study also found that overall flow state enhanced the frequency of social media use (51). However, based on the flow theory and the psychological need framework, active use is likely to be proximal to COVID-19 stress as a way of coping whereas flow is proximal to addictive SMU in the serial mediation chain. In other words, it is more likely that individuals first adopt active SMU behaviors as a result of coping with stressful events and then fall into an immersed pleasant state through repeated use, which ultimately leads to SM addiction. Research on smartphone use revealed that people who use a smartphone for entertainment and sociability, especially to fight off negative feelings are more likely to achieve flow state, which is partly supportive of the sequence (52). Future studies should explore the trajectories of these various risk factors across time to uncover the directionality of active use and SM flow.

The current study extends our understanding of how COVID-19 stress is related to addictive SMU by uncovering the mediating roles of active use and flow experience. Although a clear answer to the order of two mediators cannot be given, our results may open new avenues for the prevention, identification, and intervention of addictive SMU behaviors. During the COVID-19 pandemic, it is crucial for people who are threatened by COVID-19 stress to be aware of potential maladaptive coping strategies and to refrain from excessive SMU. Furthermore, offline support from families or communities could be provided for those in need to encourage them to solve issues in the real life instead of getting immersed in the online world. Additionally, interventions may focus on fostering intentional awareness of one's state and exercising self-control over SMU, for example, by mindfulness practice to reduce addictive SMU (53).

Inevitably, this study has several limitations. First, the reliability of active use is relatively low, which limits the interpretations of the current results. Besides, self-report active SM activities may yield measurement bias. Future studies are recommended to obtain objective data that reflects individuals' SMU behaviors, such as the number of comments, “likes,” status updates to improve the assessment accuracy (54). Second, explanations for the relationship between stress and active use are conjectural since the motivation for active use was not measured in the present study. Therefore, it is unknown for what reasons people actively use SM (e.g., for social interaction, information seeking, or escape from negative emotions) when confronted with pandemic-related stress. Third, as an intrinsic rewarding state, SM flow seems to bring about negative consequences in our study. Future research could also investigate the potential positive psychological effects and benefits resulting from the optimal experiences while using SM. Fourth, the generalizability of the current findings is limited by the comparably young sample. It is also worth noting that the study did not find any sex-related differences. Therefore, it is necessary to replicate results in specific populations (e.g., adolescence) or in more balanced age composition of the sample. Fifth, confounding variables may possibly exist and should be taken into account in future research. For example, people with pre-existing psychopathology are both susceptible to external stress (55) and addictive Internet use (56). Finally, the correlational nature of the study allows only hypothetical conclusions about the causality of the described associations. Alternative explanations for the directionality of variables are possible. For example, previous research found that increase in SMU frequency can be predicted by SM addiction, which further facilitated future active use (57). Future work is encouraged to use a cross-lagged design or intensive repeated measures (e.g., experience sampling method) of active use, flow, and addictive SMU to see, whether the results of the present study can be generalized.

## Conclusion

In sum, the study showed that COVID-19 stress was positively correlated with addictive SMU. Moreover, the relationship between COVID-19 stress and addictive SMU was significantly mediated by active use and SM flow, both individually and combined. Individuals who experienced higher level of COVID-19 stress were at a higher risk of developing addictive SMU as a result of increased level of active use and SM flow.

## Data Availability Statement

The raw data supporting the conclusions of this article will be made available by the authors, without undue reservation.

## Ethics Statement

The studies involving human participants were reviewed and approved by Institutional Review Board at Peking University, School of Psychological and Cognitive Sciences. The patients/participants provided their online informed consent to participate in this study.

## Author Contributions

NZ conceived of the present study, collected the data, conducted the statistical analyses, and drafted the manuscript. GZ supervised the study and helped to revise the manuscript. All authors read and approved the final manuscript.

## Conflict of Interest

The authors declare that the research was conducted in the absence of any commercial or financial relationships that could be construed as a potential conflict of interest.
